# Exploring the Exponentially Decaying Merit of an Out-of-Sequence Observation

**DOI:** 10.3390/s18061947

**Published:** 2018-06-15

**Authors:** Josiah Yoder, Stanley Baek, Hyukseong Kwon, Daniel Pack

**Affiliations:** 1 Department of Electrical Engineering and Computer Science, Milwaukee School of Engineering, Milwaukee, WI 53202, USA; 2 Department of Electrical and Computer Engineering, University of Michigan-Dearborn, Dearborn, MI 48128, USA; stanbaek@umich.edu; 3 HRL Laboratories, Malibu, CA 90265, USA; hkwon@hrl.com; 4 College of Engineering and Computer Science, University of Tennessee at Chattanooga, Chattanooga, TN 37403, USA; daniel-pack@utc.edu

**Keywords:** Kalman filter, out-of-sequence observation, delayed measurements, selective filtering, delayed Kalman gain

## Abstract

It is well known that in a Kalman filtering framework, all sensor observations or measurements contribute toward improving the accuracy of state estimation, but, as observations become older, their impact toward improving estimations becomes smaller to the point that they offer no practical benefit. In this paper, we provide an practical technique for determining the merit of an old observation using system parameters. We demonstrate that the benefit provided by an old observation decreases exponentially with the number of observations captured and processed after it. To quantify the merit of an old observation, we use the filter gain for the delayed observation, found by re-processing all past measurements between the delayed observation and the current time estimate, a high cost task. We demonstrate the value of the proposed technique to system designers using both nearly-constant position (random walk) and nearly-constant velocity (discrete white-noise acceleration, DWNA) cases. In these cases, the merit (that is, gain) of an old observation can be computed in closed-form without iteration. The analysis technique incorporates the state transition function, the observation function, the state transition noise, and the observation noise to quantify the merit of an old observation. Numerical simulations demonstrate the accuracy of these predictions even when measurements arrive randomly according to a Poisson distribution. Simulations confirm that our approach correctly predicts which observations increase estimation accuracy based on their delay by comparing a single-step out-of-sequence Kalman filter with a selective version that drops out-of-sequence observations. This approach may be used in system design to evaluate feasibility of a multi-agent target tracking system, and when selecting system parameters including sensor rates and network latencies.

## 1. Introduction

In many estimation applications, sensor observations or measurements are received out-of- sequence (OOS); that is, the observations arrive at the filter in a different order than they were measured. A few examples include missile guidance with delayed measurements [1], multiple unmanned vehicles [2,3], teleoperation systems [4], and networked control systems [5]. Although standard filtering techniques (such as the Kalman filter) cannot be used directly when data arrive out-of-sequence, several techniques for incorporating these delayed observations have been developed. When a delayed observation arrives at a filter, an out-of-sequence filter can use that observation to improve the accuracy of the estimate.

There are a few reasons why it may be beneficial to simply drop some of the delayed estimates instead of processing them with the OOS filter. First, optimal out-of-sequence filters must re-process all previous estimates to find the current estimate. The more an OOS observation has been delayed, the longer it will take to process. Second, approximate OOS filters (those whose runtime is constant with respect to how delayed an observation is) provide no guarantee that the delayed observation will actually improve the performance. In fact, as our simulations show, delayed observations can actually hurt their performance! For these filters, dropping a delayed observation not only reduces processing time, it also has the potential to improve the accuracy of the filter’s estimate.

### 1.1. Prior Work

Out-of-sequence observations can be incorporated optimally with the costly approach of re-processing all newer observations every time a delayed observation arrives. This requires storing all observations received in chronological order. In some cases, this is the only way to achieve this optimal result [6]. In other cases, additional assumptions can be made which simplify the out-of-sequence tracking problem [6]. For example, an algorithm proposed in [7,8] incorporates a single out-of-sequence observation optimally, assuming that the measurement time and covariance of the observation are known in advance. Even if nothing is known about the observation before it arrives, it can still be optimally fused using the A1 algorithm of [9], as long as the out-of-sequence observation arrives within the last sampling period (The last sampling period is the time between the second-most and most recently measured observations that have arrived at a filtering center).

Some out-of-sequence filters save processing time by providing an approximate rather than optimal estimate based on the out-of-sequence observations. The B1 and C1 algorithms from from [9] are examples of these. Several tradeoffs between the complexity of both storage and computation and the accuracy of the algorithm are explored in [6], including a linearly constrained optimal estimate while storing only the latest state estimate with its covariance. Three new algorithms for the general out-of-sequence filtering problem are presented in [10]. For summaries of the current literature regarding filtering techniques with time delayed measurements, see [10,11,12,13,14].

While all out-of-sequence observations can theoretically be used to improve estimation accuracy with an optimal OOS filter, if a measurement is old enough it will have an insignificant contribution to the estimate of the current state. Under certain conditions, it has been demonstrated experimentally that observations may have no practical contribution to state estimation accuracy after as few as two to five time-steps [15,16]. In such cases, it is computationally more efficient to discard observations that would provide no practical contribution. Then, one may ask what factors determine whether an observation should be discarded. A variety of experiments have demonstrated that increasing randomness in an object’s motion, decreasing observation noise, and increasing the amount of delay of an observation all decrease its contribution to estimation accuracy [15,16,17]. The experiments of [15] suggest that the impact of an observation decreases exponentially with its delay, but it is still obscure why the decrease would be exponential when the motion and measurement uncertainties increase only linearly or quadratically.

The experiments in [15,16] suggest that we should focus our attention on the ratio of an object’s motion, relative to the observations that are made of it. (This dimensionless quantity is known as the maneuvering index [6,9,18,19]). These experiments show that the maneuvering index plays a critical role in determining the value of an out-of-sequence observation. To improve the estimation accuracy and stability of an OOS filter, OOS observations can be dropped if they will not contribute to the estimate. Tasoulis et al. propose an automatic technique for determining whether to keep an observation based on a hypothesis test comparing covariances fusing all measurements with maximum lag *l* and lag l+1 [15]. The measurement is only fused if there is a significant improvement in the covariance according to a predetermined significance level. In [20], an approach is presented to determine which observations to incorporate to minimize tracking error within an average processing- time constraint.

### 1.2. Problem Statement

In summary, out-of-sequence observations are known to be of limited value if they have been delayed by too long. Dropping some old observations improves computational efficiency while maintaining nearly-optimal performance [15,16,17].

However, how do we determine when an observation has been delayed too long? Is there a way that we can gain some intuition about when we should drop an observation and when we should keep it? It is known that the value of an observation increases as the measurement time decreases, as its own observation noise decreases, as the target’s motion becomes more predictable, and as the other measurements’ observation noise increases. However, is there a way to incorporate these into a closed-form expression to estimate the impact an observation has on the accuracy of the current estimate? In this paper, we make a step toward this goal.

Our goal in this paper is to go beyond proposing yet another selective filter with an ad-hoc heuristic. We desire to provide a practical way of thinking about selective filtering that will help to explain why delayed observations behave the way they do.

### 1.3. Contributions

In particular, we would like to convince the reader that the usefulness (or merit) of a delayed observation decays exponentially with time. Traditionally, filters operate by correcting the current prediction of the state based on the error in the prediction of the observation (We explain the notation used here in Section 2.1), i.e., $\hat{x}={\hat{x}}^{p}+K\left(y-H{\hat{x}}^{p}\right)$.

This allows us to reduce the problem to the estimation of an appropriate gain, *K*. Furthermore, the gain applied to a delayed observation by an ideal filter decreases exponentially with time as we demonstrate in Section 2. For the case where the maneuverability index is very low λ≪1, the magnitude of the gain *K* is proportional to ${\left(1-\lambda \right)}^{T_{d}/\Delta T}$, where λ is the maneuvering index discussed previously in Section 1.1, $T_{d}$ is the time delay of the observation, and ΔT is the mean time between observations. That is, the gain decreases exponentially with the time since the observation (measured in terms of how many measurements have been received since that time) and increases as the maneuverability decreases.

Unless an out-of-sequence approach models this exponentially-decaying nature of *K* (whether explicitly or implicity), its performance will be hurt by old observations. As we demonstrate in Section 4, for an approximate filters, incorporating some delayed observations and dropping others can achieve better the estimation accuracy than would be achieved by incorporating either all or none of the delayed observations.

The key contributions of this paper are:

- We propose using the gain given by a simple optimal OOS filter to the delayed observation to estimate the merit of a past observation.
- For the case where the maneuverability index is very low λ≪1, this gain is proportional to ${\left(1-\lambda \right)}^{T_{d}/\Delta T}$.
- We propose that observations where the ratio of the merit to a new observation is below a fixed threshold be dropped. For a very low maneuverability target moving according to a random walk, this merit leads to a threshold of $T_{d}=\frac{\Delta T_{P}}{\lambda }$, that is, a threshold that increases linearly with the mean sampling rate and decreases linearly with the maneuverability of the target.
- Through synthetic simulations, we demonstrate that the proposed technique provides a good estimate of when observations no longer have merit across the full practical spectrum of the maneuverability index.

Although we provide an approximation of the ideal Kalman gain, we are not proposing a new OOS algorithm here. We intend this approach to be used with an existing OOS filters, although it can be used with any OOS filter available. This approach complements existing approaches by providing an alternative perspective on the weight/gain that an old observation should be given, but it does not provide a technique for estimating the full matrix gain, only a bound on the elements within that matrix.

### 1.4. Assumptions

This work is focused on a Kalman filter that is operating in steady-state. During the initial stages, when the estimate covariances are still high, it could be desirable to incorporate more delayed observations or even all of them. An assumption we make in the derivation of the delayed gain (in Section 2) is that only a single observation has been delayed. During the simulations (in Section 4) we delay all observations randomly and discuss the impact this has on the derivation. Our simplest results apply in the case where λ≪1. A similar assumption is made in [20]. This case is particularly interesting for OOS filters, because the less maneuverable a target is, the more valuable out-of-sequence measurements become. Nevertheless, this approach does not require this assumption to be made—it can be applied for any value of the parameter λ.

## 2. The Delayed Kalman Gain

In this section, we propose a Kalman gain be used as a metric of the merit of a delayed observation. In particular, we use the gain that would be given to an observation if we applied the optimal but costly approach of re-processing all newer observations every time a delayed observation arrived (The “newer observations” here are all the observations *measured after* the OOS measurement which *arrived before* it). Although the delayed Kalman gain cannot be computed exactly in practice without storing and reprocessing all measurements [6], we can obtain a good estimate of its magnitude using a formula that we present later in this section.

### 2.1. Preliminaries

For a nearly-constant velocity model, the maneuvering index (or tracking index) is defined as $\lambda =\sigma_{Q}\Delta T^{2}/\sigma_{R}$, where ΔT is the time between observations, $\sigma_{Q}$ is the standard deviation of the process noise, and $\sigma_{R}$ is the standard deviation of the measurement noise. Although the form of the maneuvering index depends on the modeling assumptions, it always includes the ratio $\sigma_{Q}/\sigma_{R}$ [6,9,21].

We use a classic state transition and observation model:

$$\begin{array}{ccc} x_{n+1} & = & Fx_{n}+w_{n}\;w_{n}∼N\left(0,Q\right)\\ y_{n} & = & Hx_{n}+v_{n}\;v_{n}∼N\left(0,R\right) \end{array}$$

where *F* is the state transition matrix, *H* is the observation matrix, *w* and *v* are uncorrelated Gaussian vectors with zero mean and covariances *Q* and *R*, respectively, $x_{n}$ is the state at the $n^{th}$ time-step, and $y_{n}$ is the observation at time *n*. (When all subscripts in an equation are the same, we omit them for simplicity.) Given this formulation, the Kalman prediction and update steps are

$$\begin{array}{ccc} {\hat{x}}^{p}{}_{n} & = & F{\hat{x}}_{n-1}\\ P_{n}^{p} & = & FP_{n-1}F^{T}+Q\\ {\hat{x}}_{n} & = & {\hat{x}}_{n}^{p}+K_{n}\left(y_{n}-H{\hat{x}}_{n}^{p}\right)\\ K_{n} & = & P_{n}^{p}H^{T}{\left(HP_{n}^{p}H^{T}+R\right)}^{-1}\\ P_{n} & = & \left(I-K_{n}H\right)P_{n}^{p} \end{array}$$

where *P* is the covariance of the estimate $\hat{x}$ and $K_{n}$ is the Kalman gain. We use a super-script *p* to denote the predictions (e.g., ${\hat{x}}^{p}$) and the absence of superscript *p* to denote the updated estimates that include the most recent observations (e.g., $\hat{x}$).

In the remainder of the paper, we refer only to the estimates of the state, $\hat{x}$, and not the state’s true value, *x*. In the sections that follow, we represent $\hat{x}$ by *x*, and ${\hat{x}}^{p}$ by $x^{p}$.

### 2.2. The Output of the Kalman Filter as Weighted Sum of Observations

The estimate produced by the linear Kalman filter is a linear combination of the observations $y_{1},\ldots ,y_{n}$ with weights (or gains) determined by the system parameters. We can see this by expanding the recursive formulas for the estimated states, both predicted $x^{p}$ and updated *x*,

$$\begin{array}{ccc} x_{n}^{p} & = & Fx_{n-1}\\ x & = & Ky+\left(I-KH\right)x^{p}, \end{array}$$

to obtain

(1) $$\begin{array}{ccc} x_{n} & = & K_{n}y_{n}+\left(I-K_{n}H\right)FK_{n-1}y_{n-1}+\left(I-K_{n}H\right)F\left(I-K_{n-1}H\right)FK_{n-2}y_{n-2}+\ldots . \end{array}$$

(2) $$\begin{array}{cc} = & K_{n}y_{n}+\sum_{j=1}^{n-1}\left\{\prod_{i=0}^{j-1}\left[(I-K_{n-i}H)F\right]K_{n-j}y_{n-j}\right\} \end{array}$$

where n>0 and we assume the initial state estimate $x_{0}=0.$ The estimate for step *n* takes the form of a linear combination of the inputs $y_{n}$, $y_{n-1}$, …, $y_{1}$ where term $y_{n-j}$ is weighted by matrix $\prod_{i=0}^{j-1}\left(\left(I-K_{n-i}H\right)F\right)K_{n-j}$.

### 2.3. Steady-State Kalman Gain

To understand how the weight, $\prod_{i=0}^{j-1}\left(\left(I-K_{n-i}H\right)F\right)K_{n-j}$, changes as the number of observations *j* increases, we focus on the situation where *Q* and *R* are constant, as the $K_{n}$’s will converge to a limiting value *K*. (In Section 3, we show numerically that this is also a good approximation when *Q* changes because observations are made non-periodically.) In order to find this value, it is necessary to solve the discrete algebraic Riccati equation (DARE),

(3) $$C^{p}=F\left(C^{p}-C^{p}H^{T}{\left(HC^{p}H^{T}+R\right)}^{-1}HC^{p}\right)F^{T}+Q,$$

where $C^{p}$ is the steady-state covariance of the prediction.

In general, the solutions to the DARE correspond to the roots of a polynomial, and thus cannot be expressed in a closed form for a sufficiently high order system (e.g., [22,23]). Nevertheless, for many applications, the DAREs have analytic solutions, and we look at two examples in the following sections.

The expanded state estimate (2) simplifies considerably when substituting the value of *K* after convergence for all the $K_{n}$’s, to

(4) $$x_{n}=\sum_{j=0}^{n-1}{\left(\left(I-KH\right)F\right)}^{j}Ky_{n-j}=\sum_{j=0}^{n-1}K_{n,j}y_{n-j}$$

where $K_{n,j}$ is the effective gain of the Kalman filter for all past observations. The effective gain $K_{n,j}$ is usually smaller than the original gain $K_{n-j}$ applied to an observation because it is reduced by the down-weighting factor ${\left(\left(I-KH\right)F\right)}^{j}$. In other words, although the ideal Kalman filtering may originally apply a high gain to an observation when it is first made, it is multiplied by gains that reduce its magnitude with each successive observation that is measured after it.

The simple linear combination (4) includes the gains that the ideal Kalman filter gives to all observations when the filter is in steady-state. This is also the gain that the optimal but costly buffered and re-filtered approach gives to a delayed observation. From (4), the gain for a delayed observation is

$$K_{n,j}={\left(\left(I-KH\right)F\right)}^{j}K$$

where the observation is delayed by *j* time-steps. Given only the time-delay of an observation, $T_{d}$, we can compute the number of time-steps $j=T_{d}/\Delta T$ between when the observation was made and our most recent estimate time, and estimate the time-delayed gain

(5) $$K_{n}\left(T_{d}\right)={\left(\left(I-KH\right)F\right)}^{T_{d}/\Delta T}K,$$

where ΔT is the time between observations. This gain, $K_{n}\left(T_{d}\right)$ is the metric that we propose for the merit of a delayed observation. We recommend selecting delayed observations whose $K_{n}\left(T_{d}\right)$ value is greater than some threshold, and rejecting the rest.

In general, $K_{n}\left(T_{d}\right)$ is a matrix quantity, and requires the solution to the discrete algebraic Riccati equation. In Section 2.5 and Section 2.6, we examine this gain for random walks and nearly-constant-velocity walks. Based on the decaying exponential bound for the function(s) making up the closed-form solution for $K_{n}\left(T_{d}\right)$ in these cases, we suggest how to select a time-delay threshold to decide whether to select a delayed observation to be included into an out-of-sequence filter. Although the general $K_{n}\left(T_{d}\right)$ cannot always be found analytically, it is still bounded by a decaying exponential envelope, as examined in the next section.

### 2.4. Exponential Behavior of the Time Delayed Gain $K_{n}\left(T_{d}\right)$

The time-delayed gain $K_{n}\left(T_{d}\right)={\left(\left(I-KH\right)F\right)}^{T_{d}/\Delta T}K$ has norms bounded by a decaying exponential function because the magnitude of all eigenvalues μ of (I-KH)F, is less than one (|μ|<1), for all systems where (F,H) is observable and (F,Q) is reachable. As a result, the impact of a time-delayed observation will decrease exponentially as the delay time increases. Numerical methods can be used to determine the eigenvalues of (I-KH)F, and the largest eigenvalue (the one with magnitude closest to unity) will play the dominant role in determining the maximum delay time.

The time-delayed gain $K_{n}\left(T_{d}\right)$ provides insights into the merit of a time-delayed observation based on the system parameters F,Q,H, and *R* as well as the delay time, $T_{d}$. In the general case, these variables are hidden in the solution to the DARE, but in many practical cases, there are closed-form solutions that provide further insights, as we shall show in the remainder of this section. In the general case, $K_{n}\left(T_{d}\right)$ can be used to decide which time-delayed observations are worth incorporating using an out-of-sequence filter.

### 2.5. Scalar Case

In many practical cases, it is possible to find a closed-form solution to the discrete algebraic Riccati equation (DARE). In the scalar case, when f=1 and h=1, the limiting value of the steady-state gain *k* (sometimes known as α) is given by [18]

(6) $$k=\frac{1}{2}\left(-\lambda^{2}+\sqrt{\lambda^{4}+4\lambda^{2}}\right)$$

where $\lambda =\sigma_{Q}\sqrt{\Delta T}/\sigma_{R}$ is the maneuvering index for a continuous random walk (also known as a Brownian motion or Wiener process), $\sigma_{Q}$ is the standard deviation of the state transition noise (in physical dimensions [length]/sqrt([time])), and $\sigma_{R}$ is the standard deviation of the observation noise (in physical dimensions [length]). All other lower-case variables are the one-dimensional counterparts of the capitalized full-matrix versions.

With this *k*, we can compute the gain of the delayed observation using the scalar form of (5)

(7) $$k_{n}\left(T_{d}\right)={\left(1-k\right)}^{T_{d}/\Delta T}k$$

Supposing that we only want to keep observations with a minimum gain of $k_{min}$, we can compute the maximum delay time to be

(8) $$T_{d}=\Delta T\frac{\log(k_{min}/k)}{\log(1-k)}$$

We use this equation in the experiments (in Section 4) to decide when to simply drop an old observation, saving computing time with only minor change in estimation performance.

When the motion model is very good (that is, when $\sigma_{Q}\ll \sigma_{R}$ and therefore the maneuvering index is small, λ≪1), the approximations k≈λ applies, and $k_{n}\left(T_{d}\right)={\left(1-\lambda \right)}^{T_{d}/\Delta T}k$. This knowledge of the low maneuvering index is significant for system design. For example, suppose that we choose to keep observations where $k_{min}/k\geq 0.6$, and suppose that the maneuvering index is λ=0.1. Then we will keep all observations whose maximum delay is $T\geq T_{d}=\Delta T\frac{\log0.6}{log(1-0.1)}=\Delta T\;4.85$, or about five time-steps.

### 2.6. Discrete White-Noise Acceleration (DWNA) Case

The DARE can also be solved in a closed-form for the common system models used to estimate positions and velocities, often called the constant velocity, the nearly-constant velocity, or the discrete white-noise acceleration model. In this model, H=[10], $F=\left[\begin{array}{cc} 1 & \Delta T\\ 0 & 1 \end{array}\right],$$Q=\left[\begin{array}{cc} \Delta T^{4}/4 & \Delta T^{3}/2\\ \Delta T^{3}/2 & \Delta T^{2} \end{array}\right]\sigma_{Q}^{2}$, and $R=\sigma_{R}^{2}.$ The steady-state Kalman gain is a function of the maneuvering index $\lambda =\sigma_{Q}\Delta T^{2}/\sigma_{R}$, ([19,21]),

(9) $$K=\left[\begin{array}{c} \alpha \\ \beta /\Delta T \end{array}\right]$$

where

(10) $$\alpha =\frac{1}{8}\left(-\lambda^{2}-8\lambda +\left(\lambda +4\right)\sqrt{\lambda^{2}+8\lambda }\right)$$

and

(11) $$\beta =\frac{1}{4}\left(\lambda^{2}+4\lambda -\lambda \sqrt{\lambda^{2}+8\lambda }\right)$$

The general form, $K_{n}\left(T_{d}\right)={\left(\left(I-KH\right)F\right)}^{T_{d}/\Delta T}K$ reduces to

(12) $$K_{n}\left(T_{d}\right)={\left[\begin{array}{cc} 1-\alpha & \Delta T(1-\alpha )\\ -\beta /\Delta T & 1-\beta \end{array}\right]}^{j}\left[\begin{array}{c} \alpha \\ \beta /\Delta T \end{array}\right]$$

The matrix (I-KH)F has eigenvalues

(13) $$1-\frac{\alpha +\beta }{2}\pm \frac{1}{2}\sqrt{{\left(\alpha +\beta \right)}^{2}-4\beta }$$

and as stated in Section 2.4, both eigenvalues have magnitude less than 1, and the matrix gain has a decaying exponential envelope.

The random-walk and position-and-velocity cases are easily extended to multiple dimensions. As long as there is no mixing between the dimensions, the formulas from these sections can be applied to each dimension independently.

## 3. Numerical Validation

In many applications where observations are delayed, the observations are also made asynchronously.

To validate the gain formulas provided in the previous section when observations are not made according to a fixed schedule, we compute these gains numerically in a scenario where observations occur according to a Poisson process. Our simulation uses a Kalman filter that maintains the delayed gain $k_{j,l}$ and predicted delayed gain $k{}_{j,l}^{p}$ of all observations. We computed these gains using Algorithm 1.

**Algorithm 1** Computation of ideal Kalman filter gains on Poisson-distributed sequence. The gains do not depend on the actual measurements, so there is no need to include them in the calculations.

$k_{1,1}^{p}=1$

for *j* from 1 to *n*
  $k_{j,0}=c_{j}^{p}/\left(h^{2}c_{j}^{p}+\sigma_{R}^{2}\right)$
  $c_{j}=\left(1-k_{j}h\right)c_{j}^{p}$
  $k_{j,l}=\left(1-k_{j}h\right)k_{j,l}^{p},\;l\in 1,\ldots ,j$
  $c_{n+1}^{p}=c_{n}+\Delta T_{n}\sigma_{Q}^{2}$
  $k_{j+1,l}^{p}=k_{j,l+1},\;l\in 0,\ldots ,j$
end


This algorithm tracks the gain (or weight) of each observation on the current estimate. The estimate $x_{n}$ can be expressed as $x_{n}=\sum_{l=0}^{n-1}k_{n,l}y_{n-l}+k_{n,n}x_{0}$ where $k_{n,l}$ is the gain of the observation delayed by *l* time-steps on the estimate at time *n*, and the prediction $x_{n}^{p}$ can be expressed as $x{}_{n}^{p}=\sum_{l=0}^{n-1}k_{n,l}^{p}y_{n-l}$+$k_{n,n}^{p}x_{0}$, where $k_{n,l}^{p}$ is the gain of the observation delayed by *l* time-steps on the prediction at time *n*. This code is used to experimentally calculate the true delayed gains that would have been applied to an estimate. Because the gains do not depend on the actual observations, there is no need to compute the observations.

When the observations are uniformly spaced, our filter computes the delayed Kalman gain $k_{n}\left(T_{d}\right)$ exactly. When observations are not uniformly spaced, the state transition noise $\Delta T_{n}\sigma_{Q}^{2}$ will change from time-step to time-step, and *k* and $k_{n}\left(T_{d}\right)$ will never converge exactly.

To apply (7) to Poisson-distributed observations, we simply compute the expected number of observations *j* that would have arrived in the time period *T* since the observation came in:

$$j\approx T/\Delta T_{P}$$

where the rate parameter of the Poisson distribution is $1/\Delta T_{P}$. In this case, the average time between observations will be $\Delta T_{P}$ in the place of a fixed time ΔT.

**Figure 1:**
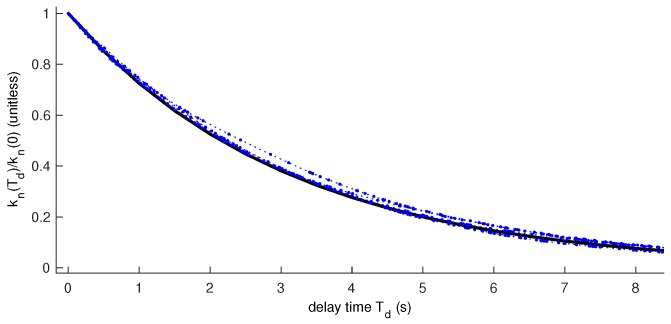
Low-λ approximation. Dashed blue lines with dots: Four experimental runs. Thick black line: ${\left(1-\frac{\sigma_{Q}\sqrt{\Delta T_{P}}}{\sigma_{R}}\right)}_{,}^{T/\Delta T_{P}}$ where $\Delta T_{P}=0.1,\sigma_{Q}^{2}=0.01,\sigma_{R}^{2}=1,h=1,f=1.$

**Figure 2:**
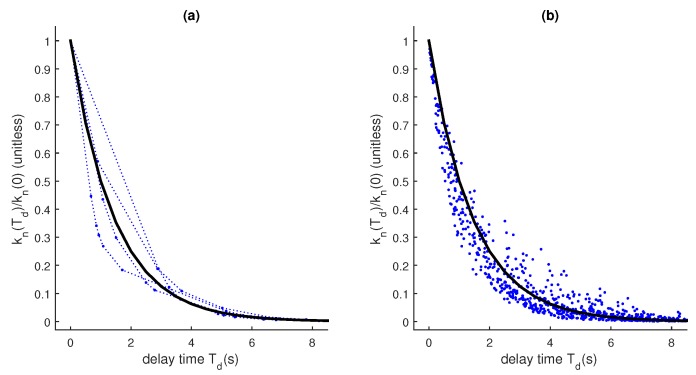
Typical-λ approximation. Dashed blue lines/dots: many runs. Thick black line: $(1-k_{n}\left(\Delta T_{P}\right)$${h)}^{T{}_{d}/\Delta T_{P}}$, where $k_{n}\left(T_{d}\right)$ is given by (6), λ=1, $\sigma_{Q}^{2}=0.5$, $\sigma_{R}^{2}=1$, h=1, f=1 (**a**) five runs; (**b**) 100 runs displayed without connecting lines.

Figure 1 and Figure 2 illustrate the result of experiments comparing $K_{n}\left(T_{d}\right)$ computed with a fixed ΔT with the actual gains computed for varying ΔT when observations arrive according to a Poisson distribution. Figure 1 illustrates a situation in which the process noise is significantly lower than the observation noise ($\sigma_{Q}\ll \sigma_{R}$). In this situation, we can use a simpler form of *k* to get a very good approximation of the weight of each observation. The figure shows the result of four example runs in this situation.

When $\sigma_{Q}$ is closer to $\sigma_{R}$, the approximation is still fairly accurate. We examined this in the experimental runs illustrated in Figure 2. We show the result of numerous runs. Because of variations in the timing introduced by the Poisson distribution, the delayed Kalman gain is not always the same. Nevertheless, $K_{n}\left(T_{d}\right)$ as computed in (5) with a fixed ΔT gives a good approximation of the actual gain given to delayed observations when they are processed in-order.

Because $K_{n}\left(T_{d}\right)$ gives a good estimate of the gain applied to an old observation even when observations are not measured periodically, it can be applied to a wide variety of systems when determining the cut-off time threshold for out-of-sequence observations.

**Figure 3:**
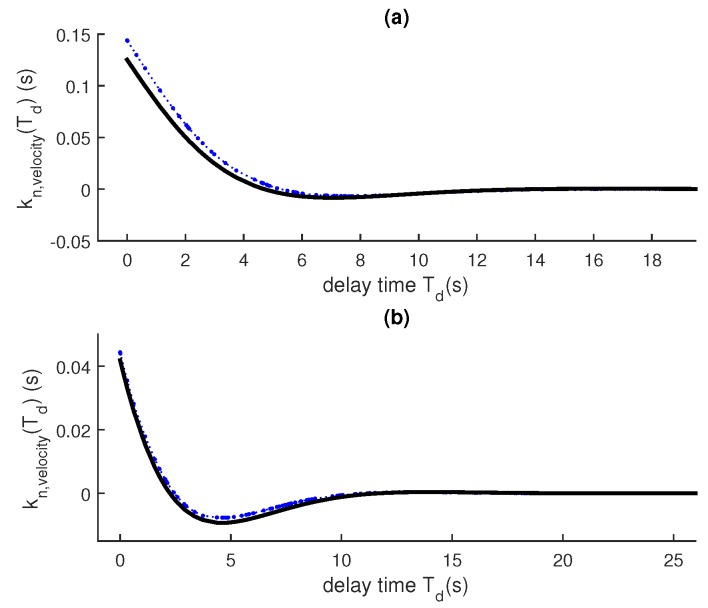
Approximation with a velocity component. Dashed blue lines/dots: One run. Thick black line: ${\left(1-KH\right)}^{T/\Delta T}K$, where *K* is found by solving the DARE. ΔT = 1/5, *Q* = 0.01$\left[\begin{array}{cc} 0 & 0\\ 0 & 1 \end{array}\right]$, *R* = 1, *H* = [1 0], $F=\left[\begin{array}{cc} 1 & \Delta T\\ 0 & 1 \end{array}\right].$ (**a**) position; (**b**) velocity.

Figure 3 shows the result of a similar experiment using a discrete white-noise acceleration model instead of a random walk.

## 4. Simulations and Discussion

### 4.1. Nearly-Constant Position (Random Walk)

The gain $K_{n}\left(T_{d}\right)$ not only provides intuition into the exponentially-decreasing value of a time- delayed observation but also can be used to set the time-delayed threshold, for example, using (8) from Section 2.5.

In this section, we run a series of experiments demonstrating that the threshold given by (8) accurately predicts which delayed observations contribute to an out-of-sequence filter’s performance.

We use a simple Kalman filter (KF) implementation that drops stale observations, an out-of-sequence filter (Bar-Shalom et al.’s A*l*1 [24]), and a *selective* A*l*1 (SA*l*1) under a variety of operating conditions. For each run, we first produced *N* random steps of of a Poisson process with rate parameter $1/\Delta T_{P}$. At each event in the Poisson process, we simulated a linear Markov process where the noise added between the steps is proportional to the time elapsed $\left(w∼N(0,\Delta t\;\sigma_{Q}^{2})\right)$. We then simulated observations delayed according to an exponential distribution with mean $\Delta T_{D}$. The observations have Gaussian noise with covariance *r* added to them.

We processed these observations with the three filters mentioned above. The KF drops all late observations, the A*l*1 incorporates all observations, and the SA*l*1 selectively incorporates observations based on the threshold $T_{stale}$ (Algorithm 2). The SA*l*1 filter incorporates an observation if its time delay satisfies $T_{d}=t_{e}-t_{m}<T_{stale}$, where $t_{e}$ is the time of the most recent estimate, and $t_{m}$ is the measurement time of the delayed observation. When the SA*l*1 incorporates out-of-sequence measurements, it uses the A*l*1 technique.

**Algorithm 2** A selective filter uses an existing out-of-sequence filter to incorporate observations that are newer than a fixed threshold.
field $T_{d}$
field oosFilter
field lastEstTime
function init($T_{d}$):
  $field.T_{d}$ = $T_{d}$
end
function update(time,observation):
  if $time-lastEstTime<T_{d}$:
    oosFilter.update(time, observation)
    lastEstTime = time
  end
end


We compare the estimates produced by each of the filters at each of the *N* time-steps with the true state at the filter’s estimate time. The three filters use exactly the same estimate time—the maximum measurement time of the observations currently received. When an out-of-sequence measurement comes in, all the filters keep their current estimate time instead of going back to the measurement time of the out-of-sequence observation. They either update their estimate by incorporating the out-of-sequence observation, or simply keep their previous estimate if they choose to drop the out-of-sequence observation. For each experiment, we performed ten runs of 5000 steps and computed the root mean square error (RMSE) of each method for each run. When computing the RMSE error, we compared the estimates with the true states at the same time.

Using (8) with a $k_{min}/k$ value of 0.6, we compute the threshold for keeping the observations shown in each figure. The value of 0.6 was selected experimentally, and shows a good match across the wide range of maneuvering indexes used here.

**Figure 4:**
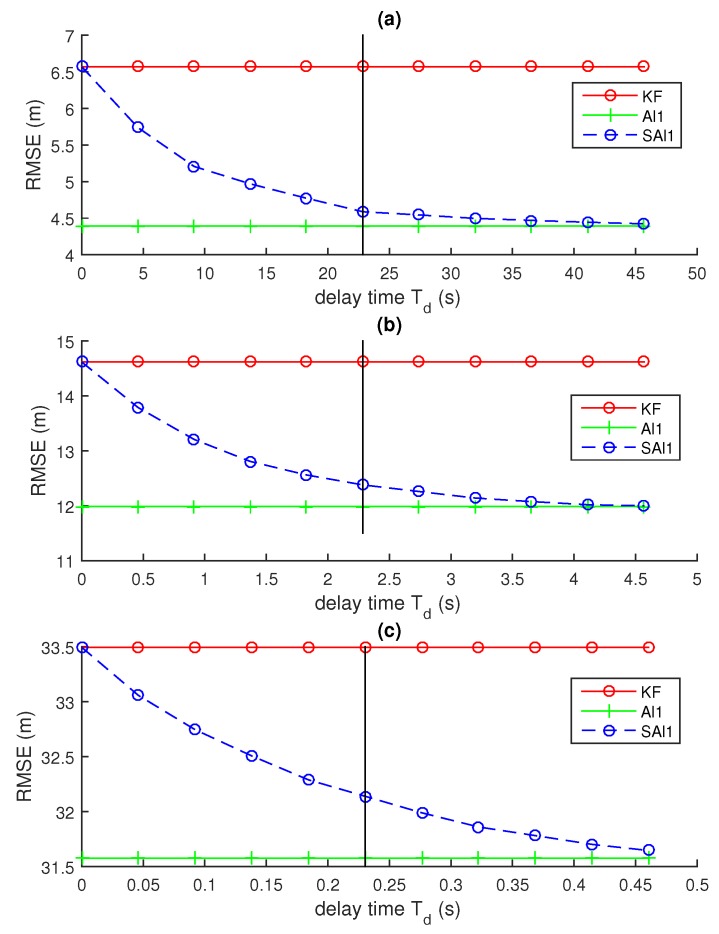
Performance of selective out-of-sequence filters as a function of the cut-off time $T_{stale}$. The vertical line represents the theoretical cut-off $T_{d}$ computed using (8) with a $k_{min}/k$ value of 0.6. (**a**) $\sigma_{Q}=0.5,\sigma_{R}=50,\Delta T_{D}=50$; (**b**) $\sigma_{Q}=5,\sigma_{R}=50,\Delta T_{D}=4$; (**c**) $\sigma_{Q}=50,\sigma_{R}=50,\Delta T_{D}=0.75$. In all runs, $f=1,h=1,\Delta T_{P}=0.2$.

Figure 4 illustrates the results of these experiments. In each case, the value of the cut-off time $T_{stale}$ accurately predicts the threshold where the performance of the SA*l*1 filter meets the performance of the A*l*1 filter, even though this threshold changes by two orders of magnitude across the examples considered. This illustrates that the time-delayed gain we propose accurately determines the threshold for selecting the most useful delayed observations.

**Figure 5:**
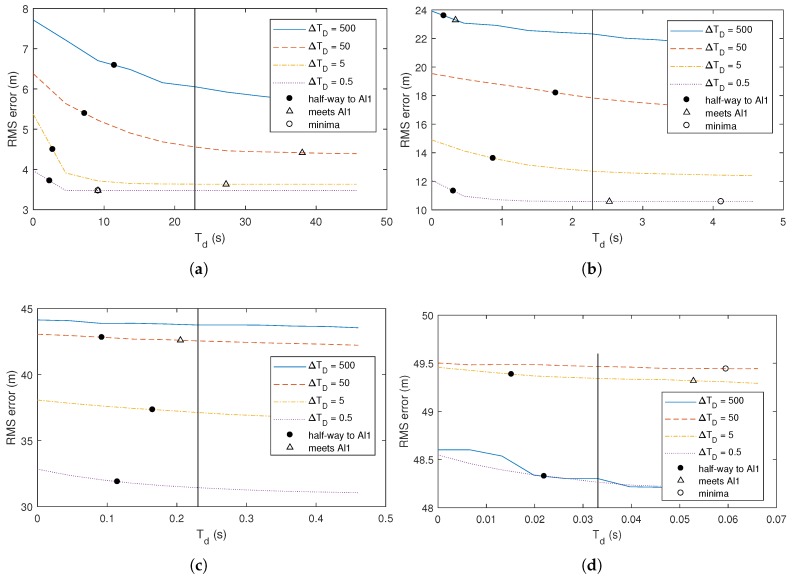
Performance of selective out-of-sequence filters as a function of the cut-off time $T_{stale}$ for a wide range of $\Delta T_{D}$ viewing a narrow time-scale. The vertical line represents the theoretical cut-off $T_{d}$ computed using (8) with a $k_{min}/k$ value of 0.6. (**a**) $\sigma_{Q}=0.5,\sigma_{R}=50$; (**b**) $\sigma_{Q}=5,\sigma_{R}=50$; (**c**) $\sigma_{Q}=50,\sigma_{R}=50$; (**d**) $\sigma_{Q}=500,\sigma_{R}=50$. In all runs, $f=1,h=1,\Delta T_{P}=0.2$.

**Figure 6:**
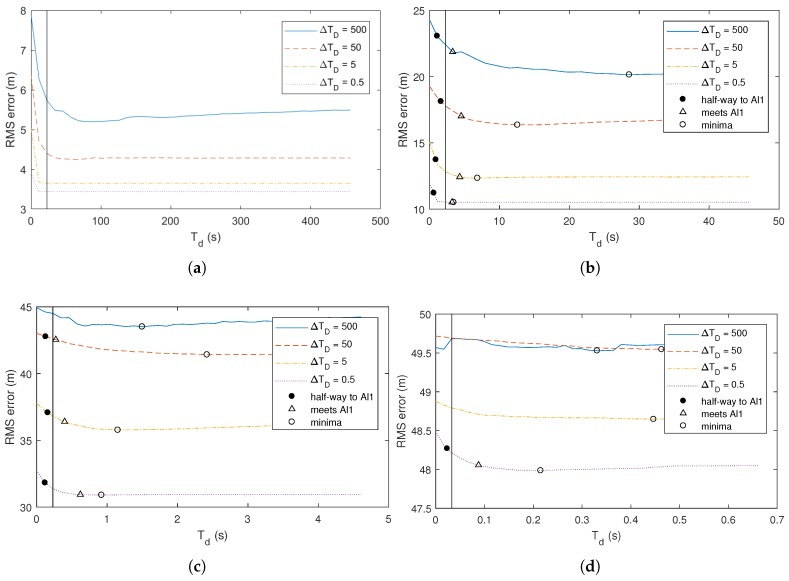
Performance of selective out-of-sequence filters as a function of the cut-off time $T_{stale}$ for a wide range of $\Delta T_{D}$ viewing a narrow time-scale. The vertical line represents the theoretical cut-off $T_{d}$ computed using (8) with a $k_{min}/k$ value of 0.6. (**a**) $\sigma_{Q}=0.5,\sigma_{R}=50$; (**b**) $\sigma_{Q}=5,\sigma_{R}=50$; (**c**) $\sigma_{Q}=50,\sigma_{R}=50$; (**d**) $\sigma_{Q}=500,\sigma_{R}=50$. In all runs, $f=1,h=1,\Delta T_{P}=0.2$.

Figure 5 and Figure 6 illustrate the same experiments as Figure 4, but take the experiments further by examining the performance of the algorithm as the mean observation delay $\Delta T_{D}$ varies across several orders of magnitude. Each curve in the figure represents a run of the experiment with a different value for $\Delta T_{D}$. The two figures show the same curves at different time-scales.

Figure 5 and Figure 6 show that the points at which the performance of the SAl1 first reaches the mid-point between the performance of the KF that drops all observations and the SAl1 that improves all observations is fairly consistent despite significant change in the mean delay $\Delta T_{D}$. It also shows that point where the SAl1 first reaches the performance of the Al1 is consistent as well.

The key thing to observe in these figures is the difference in the horizontal (time) axis between parts a, b, c, and d of the figure. Despite the maneuverability changing over three orders of magnitude, the predicted $T_{d}$ is within less than one order of magnitude of the point where the SAl1 first reaches the performance of the Al1. This ranges from dozens of time steps in Figure 5a to less than 1 time step in Figure 5d.

Figure 6 illustrates that the cut-off threshold to achieve minimum error (marked with a circle (∘) in the figure) often increases as the mean delay $\Delta T_{P}$ increases. This could seem odd, as the proposed threshold $T_{d}$ is not a function of $\Delta T_{P}$. However, as $\Delta T_{P}$ surpasses $T_{d}$, many observations are never incorporated by the filter because they have been delayed by more than $\Delta T_{P}$ when they arrive. One of the assumptions we made in the derivation of $\Delta T_{P}$ is that only one observation has been delayed, and that all the observations that were made after it have already been incorporated into the filter. When many of the observations are discarded because they come later than $T_{d}$, it changes the steady-state gain of the Kalman filter. As a result, if many observations are being discarded, the cut-off point should be determined based on the mean time between observations that are actually used rather than all those that are made. If the mean delay $\Delta T_{D}$ increases, the number of observations that are actually used decreases, increasing the mean time between measurements $\Delta T_{P}$ and the optimal cutoff $T_{d}$.

Similarly, when $\Delta T_{P}$ is significantly lower than $T_{d}$, it can be observed that the performance of the SAl1 reaches the performance of the Al1 at a time less than $T_{d}$. This is to be expected because all observations have less delay than $T_{d}$ and should all be incorporated.

### 4.2. Arrowhead Path

**Figure 7:**
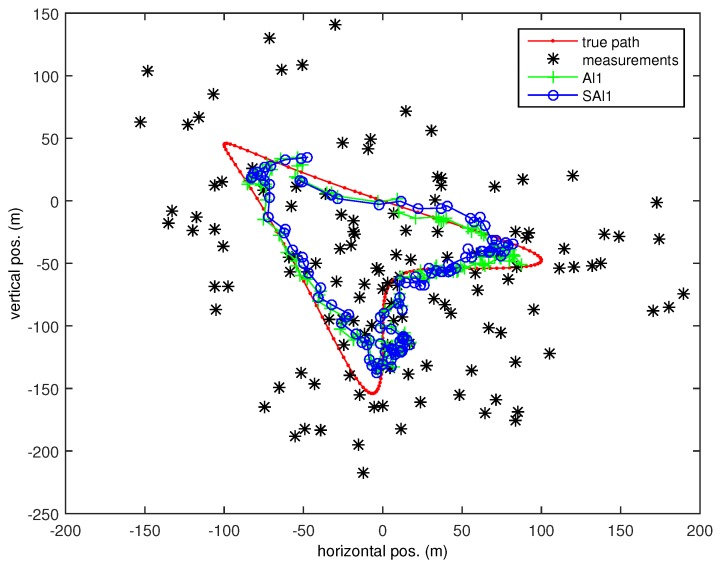
Arrowhead Path with random walk tracker.

The white Gaussian process model, while convenient, is not a realistic model for predicting target motion. To investigate selective filtering when a target is following a nonlinear trajectory, we performed an experiment with the target following a deterministic non-linear path. The path is the arrowhead given by the parametric equations

(14) $$x\left(t\right)=\left[\begin{array}{c} \rho \sin(\omega t+\sin\omega t)\\ \rho \cos(\omega t+\cos\omega t-\cos1) \end{array}\right]$$

with radius ρ=100 m, nominal speed s=5 m/s, and angular speed ω=s/ρ=0.05 rad/s (Illustrated as the red/light-gray line with dots in Figure 7).

**Figure 8:**
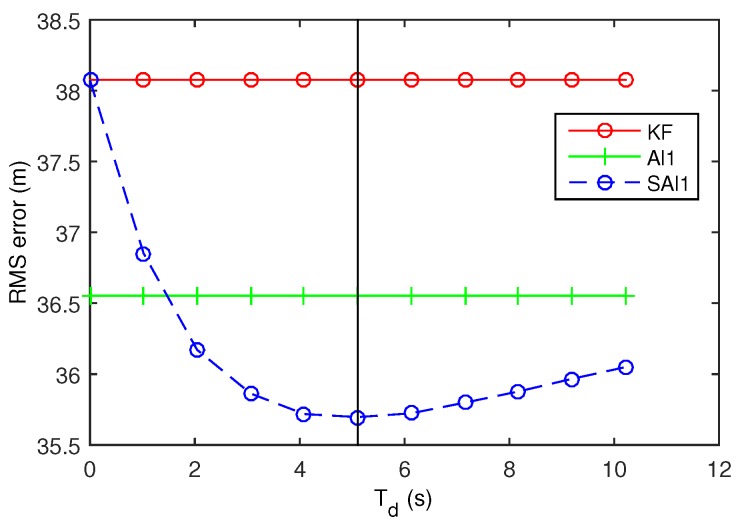
Performance of selective out-of-sequence filters for Arrowhead Path.

For tracking, we used a random walk model (without a velocity component), with the two spatial dimensions decoupled. To determine the model noise, we measured the actual standard deviation of the motion noise along each dimension, and used the maximum noise, with rounding. This resulted in a process noise model variance of q=20 m${}^{2}$/s. To generate the position observations, we added Gaussian noise with variance r=2000 m${}^{2}$. We used a regular time-step of $\Delta T_{P}=1$ s (not a Poisson process), and added delay to the observations according to an exponential distribution with mean delay $\Delta T_{D}=5$ s. With a $k_{min}/k$ value of 0.6, and from (8), the time threshold to keep a stale observation is $T_{stale}=5.11$, as illustrated by the vertical line in Figure 8. As in the previous section, we averaged the performance of the filter over 30 runs of 10,000 steps.

In these experiments, we are using an approximate out-of-sequence filter, the Al1 filter, rather than the expensive approach of buffering and re-processing observations when a delayed observation arrives. The Al1 filter is significantly faster than the buffered approach, but it does not guarantee an optimal result. Unlike the ideal Kalman filter, a delayed observation can actually hurt the performance of the Al1 filter.

**Figure 9:**
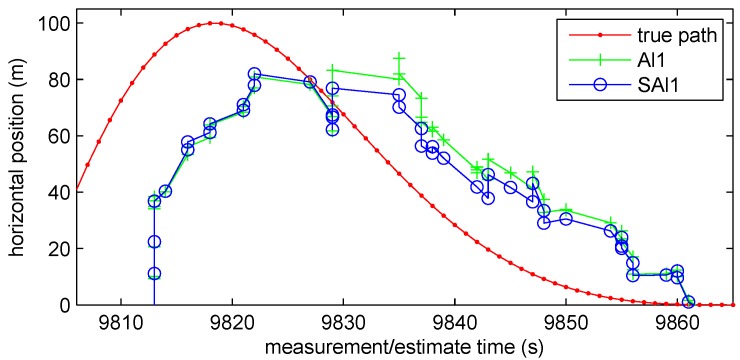
Performance of SAl1 filter compared with Al1 filter.

We see how delayed observations can hurt a filter without optimal guarantees in Figure 8 where the selective Al1 (SAl1) filter has better performance at $T=T_{stale}$ than the Al1 filter incorporating all observations. Figure 9 shows how the Al1 filter lags more as it comes around the eastern end of the track (where the horizontal position reaches its maximum), because it is suboptimally incorporating older observations that the SAl1 does not. Increasing the threshold beyond $T_{stale}$ actually hurts the performance of the SAl1 filter as it too incorporates older observations suboptimally. Contributing to the problem is the fact that the Kalman filter makes an assumption that the motion steps are random and uncorrelated when they are in fact deterministic and highly correlated.

Despite the invalid assumptions of the tracking model, our approach gives a good rough estimate of how much delay observations can have and still contribute significantly to estimation accuracy.

### 4.3. Extension to Nonlinear Filter

To investigate our approach in more general cases, we implemented an extended Kalman filter (EKF) for tracking a target that is following the arrowhead trajectory as follows

(15) $$\begin{array}{ccc} \left[\begin{array}{c} x_{k+1}\\ y_{k+1}\\ v_{k+1}\\ \theta_{k+1} \end{array}\right] & = & \left[\begin{array}{c} x_{k}+\Delta v_{k}\cos\left(\theta_{k}\right)\\ y_{k}+\Delta v_{k}\sin\left(\theta_{k}\right)\\ v_{k}\\ \theta_{k} \end{array}\right]+\omega_{k} \end{array}$$

(16) $$\begin{array}{ccc} z_{k} & = & Cx_{k}+\nu_{k} \end{array}$$

where $x_{k}={\left[\begin{array}{cccc} x_{k} & y_{k} & v_{k} & \theta_{k} \end{array}\right]}^{T}$ is the state vector at *k*, *z* is the observation vector, $\left(x_{k},y_{k}\right)$ is the location of the target, $v_{k}$ and $\theta_{k}$ are, respectively, the speed and heading of the target, $\omega_{k}∼N\left(0,Q_{k}\right)$ is a zero mean Gaussian random vector with covariance $Q_{k}$, representing the system disturbance, and $\nu_{k}∼N\left(0,R_{k}\right)$ is a zero mean Gaussian random vector with covariance $R_{k}$, representing the measurement noise. We assume that all states are completely measurable, i.e., $C=I^{n}$, where $I^{n}$ is the n×n identity matrix.

In this experiment, we have designed the EKF to drop delayed measurements if the delay time is greater than $T_{d}$. Otherwise, the EKF recursively processes them. For example, $T_{d}=0$ means the filter does not process any delayed observations and $T_{d}=\infty$ means the filter processes all measurements, and therefore, the state estimates become identical to the case with no delay.

**Figure 10:**
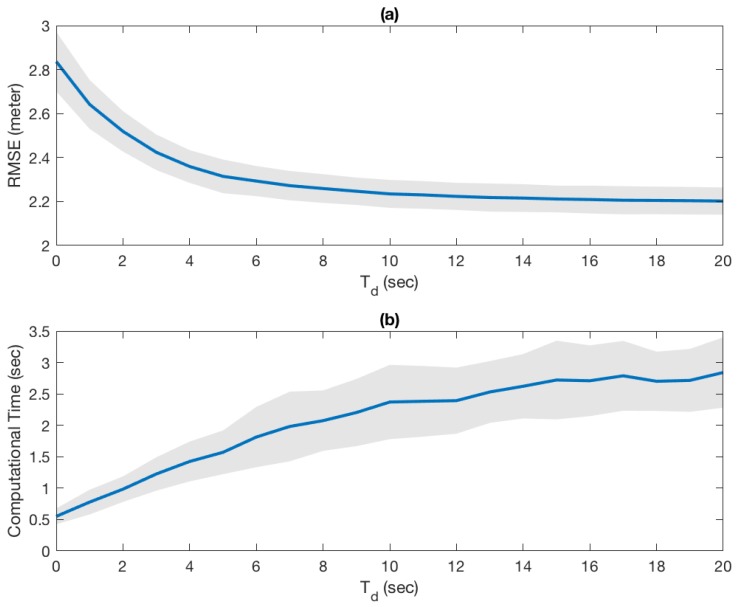
Performance of Extended Kalman fileter. (**a**) (RMSE) between the true paths and estimated trajectories with respect to the maximum delay; (**b**) Computational time with repect to the maximum delay. The gray bands indicate 1 standard deviation from the mean.

The root mean square error (RMSE) between the true paths and estimated trajectories with respect to the maximum delay ($T_{d}$) is shown in Figure 10a. The solid blue line is the average of 30 runs and the gray band indicates 1 standard deviation from the average. As it is expected, the RMSE exponentially approaches the minimum RMSE where we incorporate all delayed measurements. On the other hand, the computational time (Figure 10b) increases almost linearly, which indicates that there is not much merit to incorporate very old observations. It is evident that there is no closed-form solution like (5) in this case because the model is not linear time-invariant. Consequently, it is difficult to analytically find $T_{stale}$. However, this experiment shows that there is limited improvement on state estimates after $T_{d}>5$ and it would be safe to set the threshold time to be $T_{stale}$ = ∼5 s, which corresponds to our analysis with the linear counterpart discussed in Section 4.2.

## 5. Conclusions

This paper introduces a measure of the merit of a delayed observation based on the gain given by the ideal filter that reprocesses all newer observations every time a delayed observation arrives. We have demonstrated that this gain is a realistic estimate even when random measurement times perturb the system, and have demonstrated that this metric provides a simple and effective way to decide which observations to keep in a selective out-of-sequence filter.

It is useful to know when a delayed observation will not improve performance. The maximum delay time can be used to set the size of buffers if recent observations are stored to improve accuracy. The maximum observation delay can be used to decide whether an out-of-sequence filter will improve performance or not. Finally, the formulas provided in this paper can be used throughout the design of a tracking system system to predict how system parameters such as measurement delay, network latency, target speed, sensor accuracy, or sensing rate will impact system performance.

We believe that the intuition provided by this our approach is fundamental to the design of out-of-sequence filters. It provides critical intuition as to why some old observations do not improve performance, and others do. We hope that it will help shape the development of future OOS filters, whether they are selective or not.

Although the derivations in this paper have used linear systems, we have demonstrated that the theory here also lends intuition to the design of nonlinear variations on the Kalman filter such as the Extended Kalman Filter (EKF), Unscented Filter and, more generally, any Sigma-Point Kalman Filter (SPKF). As systems become more and more non-linear, the analysis above will become more and more of an approximation. Nevertheless, we expect that for many non-linear systems, the delayed Kalman gain is still exponentially decreasing. Investigating to what extent this is true in general nonlinear systems is the plan for our future work.
